# Effect of AlN on the Mechanical and Electrochemical Properties of Aluminum Metal Matrix Composites

**DOI:** 10.3390/ma17133258

**Published:** 2024-07-02

**Authors:** Rokaya H. Abdelatty, Ahmed Bahgat Radwan, Khaled Youssef, Muhammad Farzik Ijaz, Rana Abdul Shakoor

**Affiliations:** 1 Center for Advanced Materials (CAM), Qatar University, Doha 2713, Qatar; ra1506578@qu.edu.qa (R.H.A.); ahmedbahgat@qu.edu.qa (A.B.R.); 2 Materials Science and Technology Graduate Program, Department of Physics and Materials Science, Qatar University, Doha 2713, Qatar; 3 Department of Mechanical and Industrial Engineering, Qatar University, Doha 2713, Qatar; 4 Mechanical Engineering Department, College of Engineering, King Saud University, P.O. Box 800, Riyadh 11421, Saudi Arabia; mijaz@ksu.edu.sa

**Keywords:** composites, mechanical properties, microwave sintering, microstructure, corrosion

## Abstract

In the present investigation, aluminum metal matrix composites (AMMs) reinforced with aluminum nitride (AlN) nanoparticulates at different volumetric ratios of (0, 0.5, 1, 1.5, and 2 vol.%) were manufactured via a microwave-assisted powder metallurgy technique. The morphological, physical, mechanical, and electrochemical properties of the produced billets were examined to reflect the impact of the successive addition of AlN into the aluminum (Al) matrix. The morphological analysis revealed the high crystalline patterns of the formation of the Al-AlN composites. The microstructural analysis confirmed the presence of the elemental constituents of Al and AlN particles in the fabricated composites, showing an enhanced degree of agglomeration in conjunction with the additional amount of AlN. Positive behavior exhibited by the micro- and nanohardness was noticeable in the Al-AlN composites, especially at the ultimate concentration of AlN in the Al matrix of a 2 vol.%, where it reached 669.4 ± 28.1 MPa and 659.1 ± 11 MPa compared to the pure Al metal at 441.2 ± 20 MPa and 437.5 ± 11 MPa, respectively. A declining trend in the compressive strength was recorded in the reinforced Al samples. The corrosion resistance of the AlN-reinforced Al metal matrix was estimated at 3.5 wt.% NaCl using electrochemical impedance spectroscopy and potentiodynamic polarization. The results reveal that the inclusion of 2.0 vol.%AlN led to the lowest corrosion rate.

## 1. Introduction

Nowadays, aluminum metal matrix composites have been attracting worldwide attention due to their desirable properties, such as lightweight, high specific modulus and strength, good wear resistance, and exquisite thermal stability [1,2,3,4,5,6]. AMMCs exhibit high potential for having mechanical and physical properties that can be tailored to adequately meet the requirements of several engineering applications, including parts for aerospace vehicles, such as turbine blades, engine parts, and wings [7,8,9]. In addition, AMMCs can be utilized in a multitude of applications in the automotive industry, such as piston rings, brake drums, synchronizers, and many more [10,11]. It is commonly recognized that the selection of reinforcements highly depends on the final desired features of the produced AMMCs. Ongoing from this potential, AMMCs allow for a variety of reinforcements that can be fabricated into a preferred structure for the convenience of a certain application. Major types of reinforcing constituents are found particularly in four forms: particles, short fibers or whiskers, long or continuous fibers, and plates or flakes [2,3,4,12]. Particle and fiber reinforcements used in composites are constantly favored by researchers, considering their proficiency in providing high strength, toughness, wear resistance, and fatigue resistance to the metal matrix [13]. However, studies have focused more on exploring particle-based reinforcements owing to their low-cost modeling and production simplicity compared to other reinforcements [14].

The structural, mechanical, and tribological behavior of metal matrix composites (MMCs) is significantly influenced by the size of the reinforcing particles [5,15]. Thus, research has explicitly revealed the improved strength and modulus, increase in wear resistance, and high fatigue resistance of MMCs induced by nanoparticulate reinforcements compared to micron-sized reinforcements. This salient enhancement of MMCs’ characteristics is due to the high specific surface area that the nanoparticles acquire, which results in a higher adhesion and better interfacial interaction between the strengthening material and the metal matrix [16,17,18,19,20]. On these grounds, the fabrication of metal matrix composites has witnessed an enormous expansion in the research field for their superior mechanical and nanotribological properties. Hence, the main focus of the research was directed toward the development of AMMCs reinforced with nanosized particulates to benefit from the desirable ability of nanoparticles to produce a composite material with a better functioning performance compared to aluminum-based metal alloys. In consideration of the foregoing, aluminum nitride (AlN) ceramic nanoparticles were selected as the reinforcing additive to the aluminum metal matrix owing to its excellent inclusive properties, such as a high elastic modulus (310 GPa, 1090 °C); remarkable mechanical strength; good wear and corrosion resistance; high thermal conductivity (170–220 W/m·K, 25 °C); low coefficient of thermal expansion (4.3–4.6 × 10^−6^/K, 25–400 °C), expressing its high thermal and chemical stabilities; and high electrical resistivity (>10^14^ Ω·K, 25 °C) [21,22,23,24], as well as to benefit from the large specific surface area represented by the nanoscale particle size of AlN in an effort to optimize the mechanical properties of the aluminum (Al) alloy.

Moreover, the eventual properties of AMMCs are strongly dependent on the processing methods assigned for the manufacturing of the billets [6,25]. Different processing techniques, such as solid- and liquid-state methods, have been employed in previous studies. In particular, the solid-state fabrication method has caught the interest of researchers because of its capability to avoid any undesired interfacial reactions that might take place between the base aluminum matrix and the reinforcement [26]. Different techniques are derived from the solid-state method, such as powder metallurgy (PM), laser deposition, deformation, powder thixo-forming and eqi-channel angular pressing. Among these techniques, the PM method is the most prevalent applied method for the production of AMMCs as a result of its capacity to sustain well-dispersed reinforcements’ particles within the Al-matrix [10,26,27,28]. The production route of the PM method consists of blending, compaction, and sintering. In this project, a bidirectional microwave-assisted rapid sintering technique was adopted to produce the final homogenous and heat-treated composite product. Rapid heating, short processing time, energy efficiency, uniform and volumetric heating, and environmental friendliness are the main features that distinguish the microwave sintering technique from the available conventional sintering techniques [29,30]. Accordingly, this study was carried out to demonstrate the practicality of the microwave sintering technique on the synthesized Al-AlN composites by investigating the impact of different concentrations of AlN nanoparticles on the morphological, physical, mechanical, and electrochemical performances of the produced billets. According to the available literature, there has been no study reported so far on the synthesis of Al-AlN composites using the microwave sintering technique and highlighting their electrochemical behavior. Pure Al powder with a 99.5% purity was selected to represent the metal matrix for the purpose of exploring the actual impact of the incorporated AlN reinforcement on the base metal without the interference of additional metallic elements typically present in the Al alloys.

## 2. Materials and Experimental Details

### 2.1. Materials

**Figure 1:**
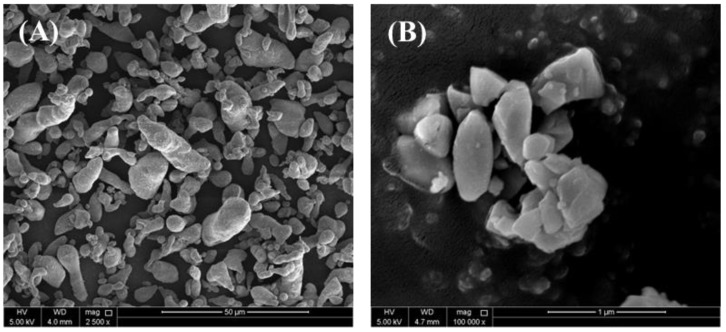
FE-SEM images of commercial powders of (**A**) pure Al and (**B**) AlN.

As-received Al powder (99.5% purity, Alfa Aesar, Tewksbury, MA, USA) with an average particle size of 10 µm and AlN powder (Sigma Aldrich, St. Louis, MO, USA) with a particle size ≤100 nm, were selected as the raw materials for the base matrix and reinforcement, respectively. The morphologies of the raw material powders are demonstrated in the form of the FE-SEM micrograph images provided in Figure 1. The volume fractions of the pure Al and AlN particle contents in the synthesized Al-AlN composite samples are provided in Table 1.

### 2.2. Composite Processing

**Figure 2:**
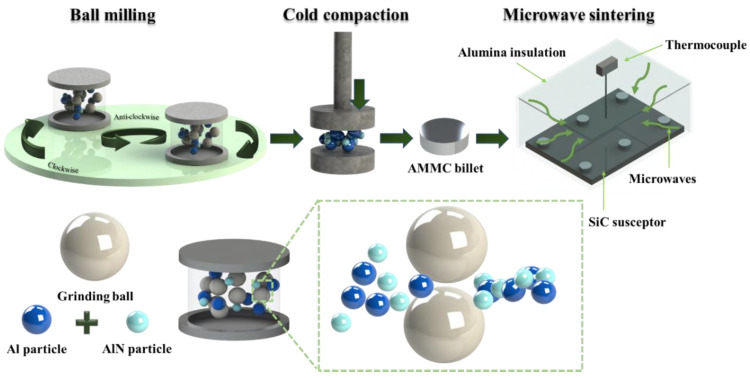
Schematic representation of the processing style of the Al-AlN composites.

For the fabrication of the Al-AlN composite samples, the PM method was implemented in this work by the ball milling and microwave sintering techniques. The stoichiometric amounts of the matrix powder (pure Al) and reinforcement powder (AlN) were weighed according to the predetermined volumetric ratios of the Al and AlN powders in Table 1. The quantifying process was performed carefully using an analytical balance (Sartorius, ENTRIS64-1S, Lower Saxony, Germany). Then, the feed materials were introduced to the planetary ball mill, a PM200 (RETSCH 20.640.0001, Haan, Germany), to produce a uniformly distributed mixture of Al metal matrix and AlN reinforcement. This step was executed under atmospheric conditions at a rotational speed of 200 RPM in reverse directions with a specified interval time of 10 min for each clockwise and anticlockwise direction for a duration of 2 h with the aim of achieving a homogenously dispersed nanoparticles of the reinforcement within the base matrix. About 1.0 g was subtracted from the uniform blended mixture of powders to be transformed into a green cylindrical form of billets by compressing them using a cold-compaction process under an applied uniaxial pressure of 50 MPa with a holding time of 1 min. Each specimen represented the exact amount of the inserted AlN reinforcement into the matrix, as per the data in Table 1. The sample’s dimensions were 13 mm in diameter and 3 mm in thickness. Identical compacting conditions were used to compact the pure aluminum in the absence of the blending step. Next, the green billets were sintered using a hybrid bidirectional microwave-assisted rapid sintering furnace purchased from VB ceramic furnace (VBCC/MF/1600 °C/14/15, Chennai, India). The specimens were contained in an insulated cubicle alumina chamber. The function of the alumina insulator is to sustain the heat generated inside the specimens. The specimens were placed directly making contact with the silicon carbide susceptor plates to accelerate the heating rate of the green billets by heating the samples from the exterior to the core of the composite. The temperature of the sintered samples was measured using a contactless IR sensor. The operational parameters of the microwave sintering furnace were set at the optimized conditions of a 550 °C sintering temperature and 30 min soaking time with a 10 °C/min heating rate. The cooling mechanism involved leaving the specimens to naturally reduce in temperature reaching room temperature conditions. Figure 2 depicts a schematic representation of the processing stages for the development of the Al-AlN composites by an optimized route of the working conditions.

### 2.3. Characterization of Composites

Different evaluation assessments were used for the investigation of the properties of the prepared microwave-sintered Al-AlN composites via powder metallurgy style. Grinding, polishing, and etching techniques were necessary for the preparation of the metallographic specimens, which were subjected to a structural assessment. The morphological analysis of the sintered Al-AlN composite samples was explored using a field emission scanning microscope (FE-SEM) (SEM-FEI Nova NanoSEM 450 FE-SEM, Hillsboro, OR, USA) equipped with energy-dispersive X-ray spectroscopy (EDS) (Bruker SDD-EDS, Coventry, UK) for the compositional characteristics of the developed composites. The microstructural investigation was conducted on the etched samples to clearly observe the AlN phase. The microstructural micrographs were taken using an optical microscope (OLYMPUS BX53M light microscope) and FE-SEM machine with elemental mapping features. An X-ray diffractometer (XRD) (PANalytical X’pert pro, PANalytical B.V., Almelo, the Netherlands) was used for the phase evolution analysis of the developed composites. The XRD spectra were identified at a scanning rate of 1.5°/min and a step size of 0.013° in the 2θ range of 20–90°. The density analysis was performed as a reflection of the physical properties of the synthesized Al-AlN composites based on Archimedes’ principle using the density kit analytical balance (Sartorius YDK03, Göttingen, Lower Saxony, Germany) with a precision of ±0.0001 g, where the water temperature was tracked throughout the entire analysis. The surface topography of the produced specimens was analyzed through atomic force microscopy (AFM) (MFP-3D AFM, Asylum Research, Oxford, UK). Vickers microhardness tester (FM-ARS9000, MKV-h21, Tokyo, Japan) and nanoindenter tester (MFP-3D Nano Indenter, Asylum Research, Oxford, UK) were both utilized in the evaluation of the hardness behavior of the prepared Al-AlN composites. Microhardness values were measured under an optimum applied load of 5 gf for 10 s, whereby five iterations were carried out for each sample to obtain reproducible data. A maximum load of 1 mN was applied to the constituted composites for the nanohardness computation under a retention time of 5 s at the highest load. Furthermore, the ultimate compressive strength (UCS) of the specimens were measured with the help of a universal testing machine (Lloyd, USA-LR50Kplus, Sussex, UK), under an engineering strain rate of 0.6 mm/min. The geometrical dimensions of the subjected samples were 13 mm in diameter and 3 mm in thickness. The stated values were an average of 4 conducted trials. All mechanical analyses were performed under ambient meteorological conditions. The fractured surfaces of the Al-AlN composites were observed using a field emission scanning electron microscope (FE-SEM). The electrochemical impedance spectroscopy (EIS) on the aluminum composite samples was recorded using a Gamry electrochemical workstation (Reference 3000, Gamry Instrument, Warminster, PA, USA), over a frequency range from 0.01 Hz to 100 kHz with an amplitude signal of 10 mV. The electrochemical responses in different parts were characterized by an electrochemical cell with a three-electrode device containing 3.5 wt% NaCl solution. The circuit potential was tested first, and the test duration was 30 min.

## 3. Results and Discussion

### 3.1. Morphological Analysis

**Figure 3:**
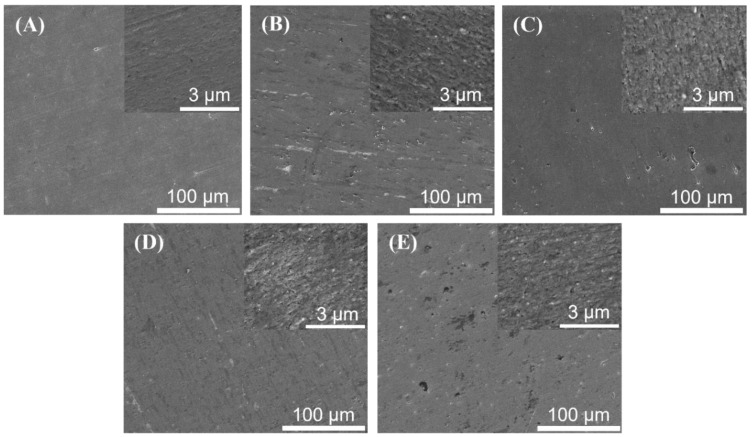
FE-SEM micrographs: (**A**) pure Al; (**B**) Al-0.5 vol.% AlN; (**C**) Al-1.0 vol.% AlN; (**D**) Al-1.5 vol.% AlN; (**E**) Al-2.0 vol.% AlN composites.

**Figure 4:**
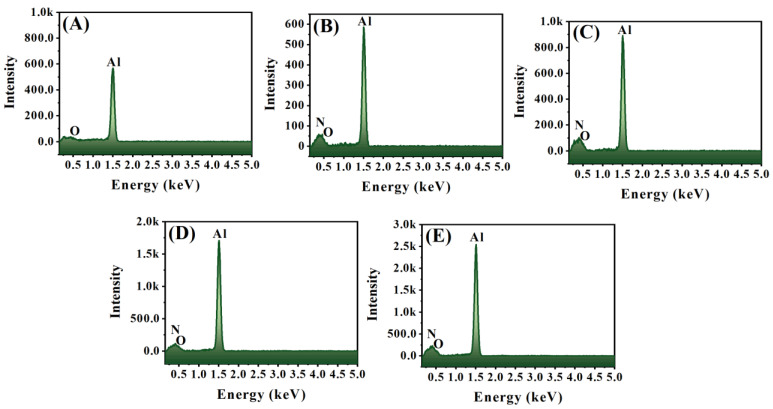
EDS analysis of (**A**) pure Al matrix; elevated concentrations of AlN-reinforced Al metal for the (**B**) Al-0.5 vol.% AlN, (**C**) Al-1.0 vol.% AlN, (**D**) Al-1.5 vol.% AlN, and (**E**) Al-2.0 vol.% AlN composites.

The surface morphologies of the sintered pure Al-matrix and Al-AlN composites containing disparate volume contents of AlN nanoparticles were studied by FE-SEM, as specified in Figure 3. Figure 3A shows the surface structure of the consolidated pure Al-matrix, indicating the absence of inclusions of AlN, which is also confirmed in the magnified inset image at a 3 µm scale. Figure 3B–E reveal the surface configuration of the fabricated Al-AlN composites with the inclusion of the reinforcing additives of AlN nanoparticles in the aluminum metal matrix. The micrographs illustrate the presence of the disseminated greyish-white particles corresponding to the AlN reinforcement, distributed evenly within the Al metal matrix which is displayed in the dark grey region. In addition, the increment in the added volume fraction of the nanosized AlN particles embedded in the aluminum matrix can be detected in the magnified pictures inserted in the corresponding images in Figure 3B–E. This is consistent with the FE-SEM images reported in previous studies conducted on Al-AlN composites [31,32]. Moreover, minor voids were spotted with the incorporated amount of AlN nanoparticles due to the high tendency behavior of the reinforcement particles to agglomerate and, therefore, initiating some pores within the composite sample. This undesired act of agglomerations is attributed to the nanosized nature of the AlN particles, whereby the large surface-area-to-volume-ratio of the nanoscale particles leads to strong adhesion among the particles owing to the Van der Waal’s forces, resulting in agglomeration formation, particularly at higher added concentrations [33,34,35]. However, this behavior was observed in a few regions only, especially at the highest concentration of AlN in the Al-matrix, as in (A5) of Figure 3E. The EDS surface analysis shown in Figure 4 was performed to examine the presence of Al and N as the constituting elements of the developed composites. Figure 4A represents only the spectrum of the Al element in the pure Al-matrix in sample A1. In Figure 4B–E, the spectra of the N element indicate that the existence of the AlN reinforcements within the Al-matrices are prominent at the different included volume fractions. The EDS results confirm the elemental constitution of Al and N in the synthesized composites without the detection of any impurities.

**Figure 5:**
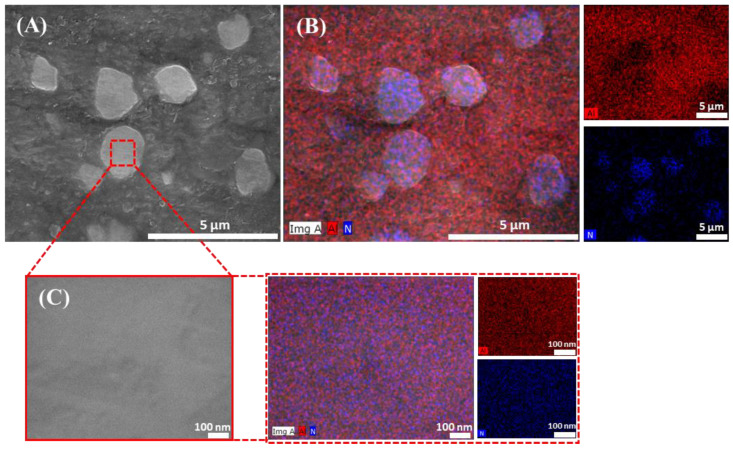
Surface analysis of the consolidated microwave-hybrid-sintered Al-2.0 vol.% AlN composite: (**A**) FE-SEM image; (**B**) elemental mapping images of the scanned SEM area; (**C**) FE-SEM magnified spot analysis of the AlN reinforcement particle with its elemental mapping images.

For further clarification of the homogeneity of the consolidated Al-AlN composites, SEM in conjunction with elemental mapping assessments was carried out on sample A5 containing 2 vol.% AlN (see Figure 5). As can be seen in Figure 5A, the AlN particles were unevenly dispersed within the Al-matrix due to their high tendency to agglomerate, especially at high concentrations.

Figure 5B depicts elemental mapping images of the same area subjected to SEM. The images clearly confirm the presence of the constitutional elements of Al and N used in the preparation of the consolidated Al-2.0 vol.% AlN composite with the absence of any secondary phases. Moreover, the N element distribution within the Al-matrix was spotted at distinctive areas, indicating the presence of AlN reinforcement despite favoring the formation of agglomerations such as in the shape of ultrafine particles, as presented in the figure. A magnified SEM image was taken from a single AlN particle to display the equal distribution of Al and N elements within the particle from the elemental mapping analysis, as presented in Figure 5C.

### 3.2. Microstructural Analysis

**Figure 6:**
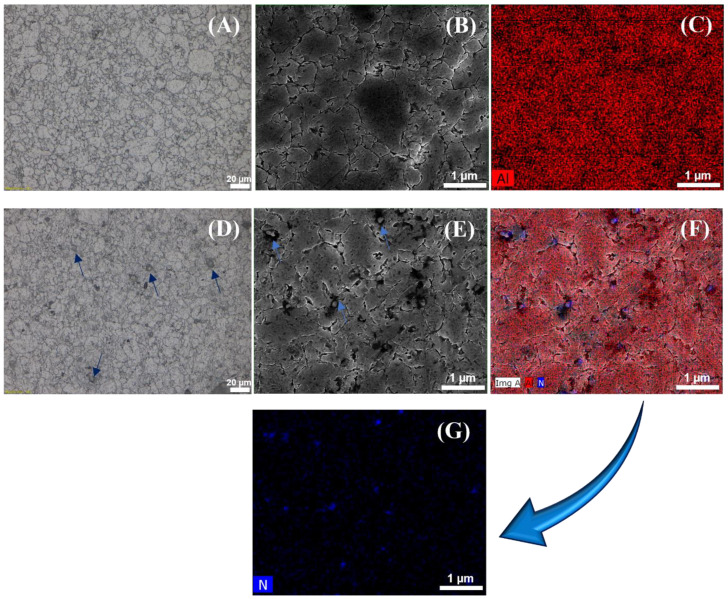
Microstructure of the consolidated microwave-hybrid-sintered pure Al sample: (**A**) optical microscopy image, (**B**) FE-SEM image, and (**C**) elemental mapping; Al-2.0 vol.% AlN composite sample: (**D**) optical microscopy image, (**E**) FE-SEM image, and (**F**) elemental mapping for aluminum; (**G**) elemental mapping for nitrogen.

To better understand the microstructures of the developed composites and pure Al metal, optical microscope, FE-SEM, and elemental mapping analyses were carried out on the synthesized (A1) and (A5) samples as demonstrated in Figure 6. Figure 6A,D show the optical microscopy images of the (A1) and (A5) samples, respectively. The presence of dark greyish particles was only visible in the (A5) sample in contrast to (A1), as indicated by the dark blue arrows. As these particles have been distributed in all regions of the matrix, a few agglomerations sites were also observed. Similar areas to Figure 6A,D were scanned by FE-SEM, producing SEM images in Figure 6B,E. The same findings were obtained, where dark greyish particles were observed along the matrix as indicated by the light blue arrows in Figure 6E. In this case, it was necessary to run an elemental mapping analysis on the same areas of the SEM images to identify the present phases in the samples. Hence, Figure 6F,G provide the elemental mapping results of the combined Al and N elements, and it was clear that the N element was spotted in the same regions of the dark greyish particles and was accumulating in some areas within the matrix, as specified in Figure 6G. However, the pure distribution of only the Al metal element is found in Figure 6C without the existence of any undesired phases. These results match the outcomes of previously reported data [36].

### 3.3. Structural Analysis Using XRD

**Figure 7:**
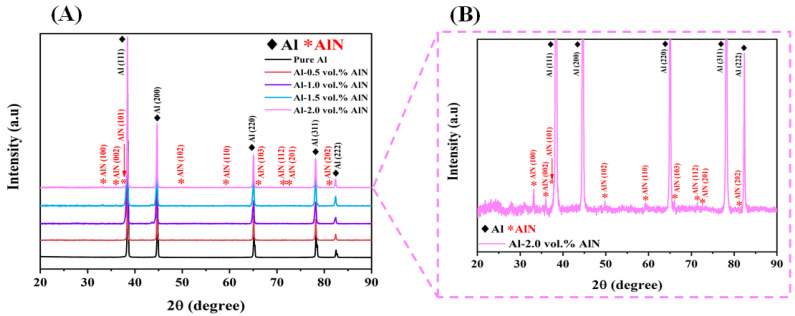
XRD spectra: (**A**) synthesized Al-AlN composites; (**B**) enlarged pattern of the Al-2.0 vol.% AlN composite.

Figure 7 represents the phase identification of the microwave-sintered pure Al and developed Al-AlN composite samples, including various volume contents of the AlN nanoparticles. The diffraction peaks of the Al-matrix at 38.5°, 44.8°, 65.1°, 78.2, and 82.5° correspond to the planes (111), (200), (220), (311), and (222), which are indexed to the ICDD database (reference code: 00-001-1180), while the AlN phases were allocated at 33.2°, 36°, 37.9°, 49.8°, 59.3°, 66°, 69.7°, 71.4°, and 81° coordinating with the planes of (100), (002), (101), (102), (110), (103), (112), (201), and (202). The XRD diffraction patterns proved the presence of the fundamental phases of the Al-matrix and AlN reinforcement nanoparticles, respectively. A sharp and high-crystallinity structure can be observed through the prominent diffracted peaks of the Al-matrix in its pure form and its composite phase. It can be clearly seen that the intensity of the Al peaks is high as a result of the immensely added concentration of Al in the Al-AlN composites. In addition, Figure 7 reveals the low intensity of the detected peaks of the AlN reinforcement due to the low content of AlN nanoparticles in the matrix. Nevertheless, the increment in the intensity of the AlN peaks starts to become more noticeable in the XRD spectra with the inclusion of higher AlN portions within the Al-matrix, reaching the highest content of 2 vol.% AlN in the Al base metal, which is compatible with earlier reported studies [31,37]. The presence of Al and AlN phases at the specified locations is evidence of the formation of Al-AlN composites and the successful implementation of the adopted powder metallurgy method and microwave sintering synthesis technique. Figure 7B demonstrates the expanded XRD pattern of sample A5 to spotlight the presence and the crystallinity of the AlN peaks. Furthermore, it reveals the absence of contaminations or other phases in the fabricated composites confirming the results of the EDS and elemental mapping.

### 3.4. Density and Porosity Analysis

**Figure 8:**
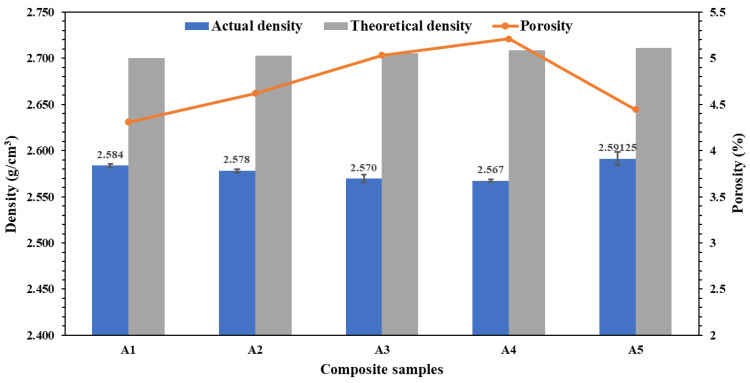
Variations in the actual and theoretical densities of the sintered Al-AlN composite samples along with their porosity measurements.

This analysis was executed to examine the physical properties of the final developed Al-AlN composite billets with the microwave sintering technique by studying the influence of the successive additions of different volume concentrations of AlN nanoreinforcement to the Al-matrix material. Figure 8 displays the relationship between the measured density, based on the displacement method of the Archimedes’ principle, and the calculated theoretical density of the pure Al billet and the composite samples, which depends on the volumetric ratio of the matrix metal to the reinforcement material, using the rule of the mixture. It can be noticed from the graph that the actual density of the Al-matrix composites has decreased with the addition of consecutive volumetric concentrations of AlN nanoparticles in the base metal starting from 2.583 g/cm^3^ in the (A1) sample and reaching to the lowest density value at 2.567 g/cm^3^ in the (A4) sample. However, a sudden improvement in the actual density was noticed at the highest volume fraction of AlN in the sample (A5), where it reached 2.591 g/cm^3^. The introduction of the AlN nanoparticles to the base matrix resulted in a decline in the actual density behavior of the composite as anticipated because of the high reactivity nature of the nanoscale size of the AlN particles, leading to the slight formation of agglomerations within the composite and, therefore, influencing the density of the composite samples by reducing it [38]. However, at the highest volume content of the AlN reinforcement, the actual density of the composite increased, which was mainly because of the increased number of AlN nanoparticles within the system. According to the fact that the theoretical density of the AlN (3.26 g/cm^3^) was higher than that of the pure Al metal (2.7 g/cm^3^), it functioned as the dominant factor for the increase in the bulk density of the composite, since the amount of AlN nanoparticles was added at the expense of the amount of Al. These outcomes are in parallel with a study by Oveisi et al. [39]. The deviation between the actual densities and the theoretical densities of the composites presents the porosity analysis. Contrary to the density behavior, the measured porosity of the sintered AlN composites was found to gradually increase after the blending of the AlN nanoparticles to the Al-matrix, from the pure Al metal at 4.31% to its highest value in the sample (A4) at 5.21% and then falling upon reaching the highest volume content of AlN in the sample (A5) to 4.44%.

### 3.5. Microhardness and Nanoindentation Analyses

**Figure 9:**
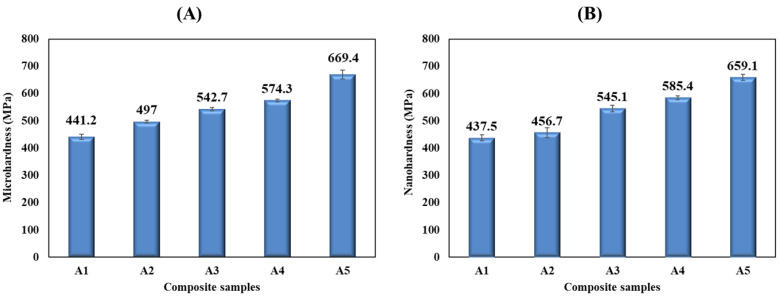
(**A**) Microhardness and (**B**) nanohardness values of the fabricated Al-AlN composite samples.

The hardness of the synthesized Al-AlN composites was investigated using a Vickers microhardness tester, and the results are demonstrated in Figure 9A. As indicated in the graph, a gradual increment in the hardness values of the consolidated billets was observed with the progressive inclusion of the AlN-strengthening phase into the (Al) base metal, reaching its optimum value at 669.4 ± 28.1 MPa, as represented by the (A5) composite sample, which provided an enhancement of the microhardness of ≅ 52% compared to the (A1) sample, which had the lowest microhardness value at 441.2 ± 20 MPa. Each composite sample was subjected to the testing of different spots, for which a very low deviation among the readings was observed, and judging by the insignificant error bars obtained by each hardness measurement, as displayed in Figure 9A, a decent distribution of the AlN reinforcement particles was attained regardless of the few aggregated particles of the AlN phase. The positive behavior of the microhardness values exhibited by the composite samples can primarily be attributed to the existence of hard AlN nanoparticles within the system of the (Al) base metal, which is a well-known dispersion hardening mechanism [40]. In addition, the improvement in the hardness after the addition of the reinforcement particles can also be supported by the rule of the mixture, as stated below [41]:

(1) $$H_{c}=H_{m}F_{m}+H_{r}F_{r}$$

where the hardness of the composite, matrix, and reinforcements is denoted by $H_{c}$, $H_{m}$, and $H_{r}$, respectively, and the volume fractions of the matrix and reinforcement are denoted by $F_{m}$ and $F_{r}$, respectively.

A nanohardness analysis was conducted as part of the conformity tests on the microhardness results. The analysis produced an outcome on the composites’ hardness, as illustrated in Figure 9B. As it is clear from the graph, the addition of the AlN reinforcement in the material composites influenced the hardness of the composite samples by increasing it. The lowest hardness value was reported for the pure Al sample (A1), with a value of 437.5 ± 11 MPa. However, after the inclusion of the AlN in the matrix, the composites’ hardness improved, reaching its highest value at 659.1 ± 11 MPa in the highest volume content of AlN reinforcement, the (A5) composite sample. These findings are in agreement with the microhardness results reported earlier in this study.

### 3.6. Compressive Analysis

**Figure 10:**
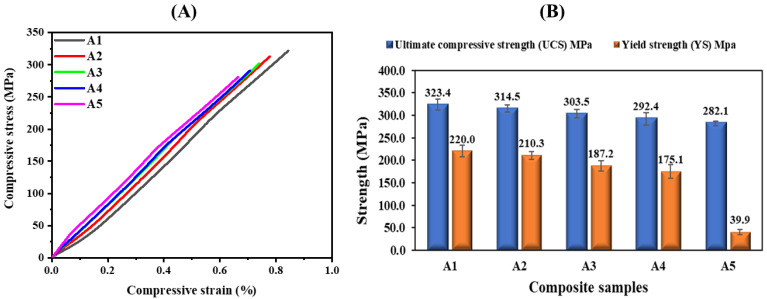
(**A**) Stress–strain curves under compressive loading; (**B**) ultimate compressive strength (UCS) and yield strength (YS) values of the fabricated Al-AlN composite samples.

A compressive strength analysis was implemented in this study to comprehend the strength behavior of the bulk pure Al matrix and the Al-AlN fabricated composites at different volume fractions. Figure 10A reveals the compressive stress/strain curves of the tested Al-AlN composite samples. Figure 10B presents the ultimate compressive strength (UCS) and the corresponding yield strength (YS) of the tested Al-AlN composite samples, with the graph clearly exhibiting the maximum obtained UCS and YS results for the pure Al matrix with values of 323.4 ± 6 MPa and 220 ± 7 MPa, respectively. However, with the blending of the AlN nanoparticles in the Al matrix, the UCS and the YS values dropped, reaching their lowest values with the highest AlN content in the Al matrix for the sample (A5), indicating 282.1 ± 2 MPa and 39.9 ± 3 MPa at a 0.2% offset, respectively. This terminal value of the UCS was noticed at a failure strain of 0.66%. The reduction percentage of the UCS in the final tested sample (A5) compared to sample (A1) was evaluated as 12.78%.

The compressive strength is strongly influenced by the even dispersion of the strengthening phase within the matrix and the bonding strength of the included nanoparticles in the matrix, which is a well-known dispersion-strengthening mechanism [42]. Hence, the observed undesired decline in the compressive strength is mainly attributed to the slight formation of AlN agglomerations within the matrix. These AlN agglomerations typically reduce the interfacial area between the reinforcement and the matrix inducing a deterioration in the overall compressive strength of the composite [43]. Moreover, the presence of pores within the matrix due to the overlapping of the AlN particles, negatively impacts the strength of the material, because it will lead to crack initiation when the composite is subjected to compressive loading; thus, it will diminish the interfacial energy bonding between the reinforcement and the matrix particles, not to mention that it will suspend the mechanism for the load transfer of the matrix particles to the reinforcing particles, and, as a result, decrease the compressive strength of the developed composite samples [44]. These findings have been discovered to be much improved over those resulting from a liquid metallurgy technique study reported by Fale et al., despite the decline in the ultimate compressive strength’s trend [42].

### 3.7. Fractography Analysis

**Figure 11:**
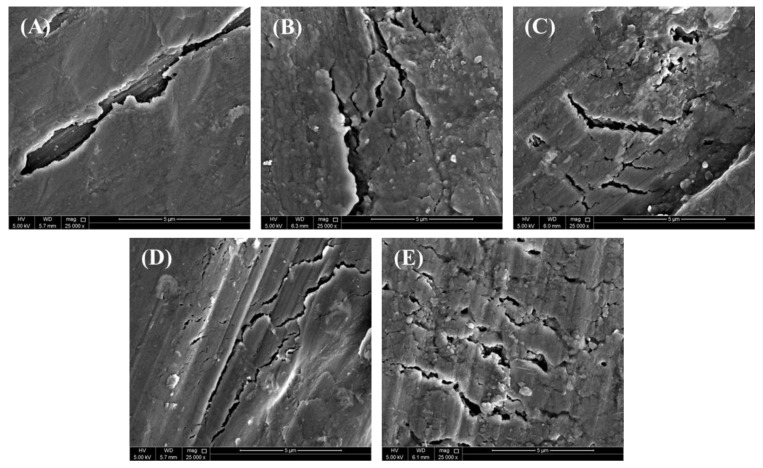
FE-SEM micrographs demonstrating compression fracture: (**A**) pure Al; (**B**) Al-0.5 vol.% AlN; (**C**) Al-1.0 vol.% AlN; (**D**) Al-1.5 vol.% AlN; (**E**) Al-2.0 vol.% AlN composites.

Figure 11 represents SEM images of the fracture morphology of the unreinforced Al billet and AlN-reinforced Al composites under compressive loading. The given images reveal the shearing mode fracture on the surface of the prepared Al matrix and Al-AlN composites when exposed to compressive loading. The SEM images clearly show the compressive deformation in the shape of cracks initiated within the billets at an angle of 45° with respect to the compressive loading axis, exhibiting severe plastic deformation behavior. The cracks are found to be more prominent in the Al-AlN composites due to the work-hardening effect.

### 3.8. Electrochemical Impedance Spectroscopy (EIS)

**Figure 12:**
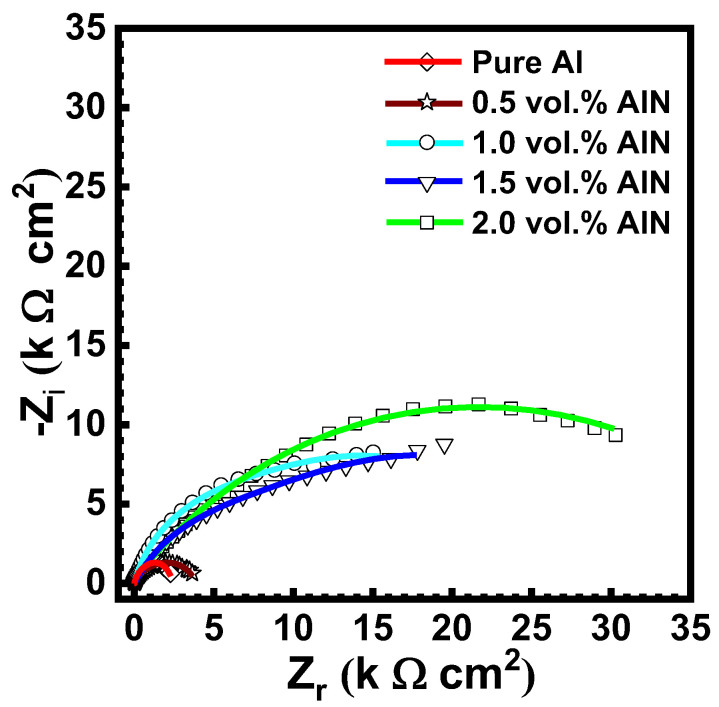
Nyquist plot of the Al matrix before and after inclusion of AlN (0, 0.5, 1.0, 1.5, and 2 vol.%) in 3.5% NaCl solution at 25 °C.

**Figure 13:**
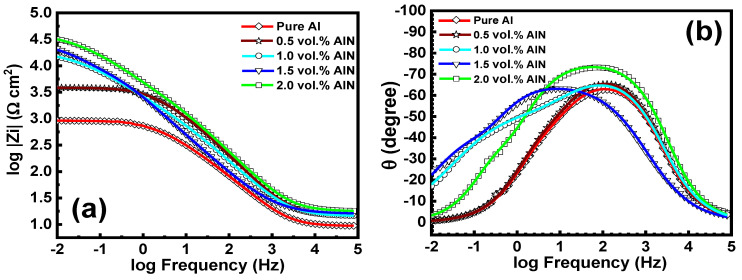
Bode plots of the Al matrix in saline water: (**a**) changes in magnitude; (**b**) phase angle as a function of frequency (Hz).

**Figure 14:**
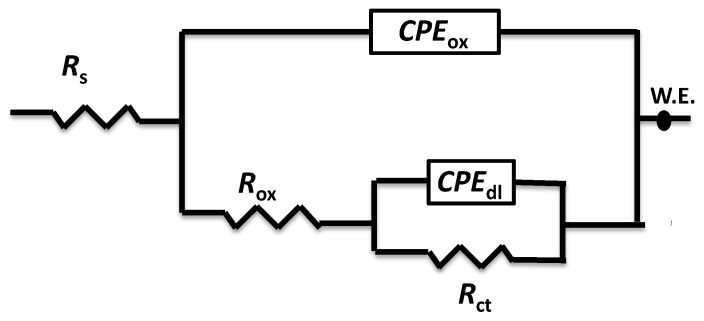
The equivalent circuit used to fit the Al-AlN composites.

Figure 12 and Figure 13 display the Nyquist and Bode blots of the measured data of the aluminum metal matrix composites in 3.5 wt.% NaCl. The experimental impedance values were fitted employing the equivalent circuit in Figure 14, and the data are reported in Table 2. The electrochemical parameters attained from the impedance diagrams are also grouped in Table 2. R_s_ represents the solution resistance at high frequency, R_ox_ is the oxide layer resistance, R_ct_ is the charge transfer resistance at low frequency, and C_dl_ is the double-layer capacitance. The constant phase element (CPE) substituted the ideal electrical capacitance in the equivalent circuit. A nonhomogeneous surface of the Al matrix can better be described by this element, which has the following impedance [45,46]:

1/ZCPE = q_o_ (jω)^α^ (2)

where q_o_ (s. Ω^−1^) equals the admittance (1/|Z|) at ω = 1 rad/s, ω is the angular frequency of the AC signal (1/rad), and α is the CPE exponent. When α equals 1, the CPE behavior approaches that of an ideal capacitor.

**C_dl1_** is the double-layer capacitance constant that can be clarified employing the following expression [47]:

(3) $$C_{dl}=\sqrt[{n}]{\frac{Q}{R^{\left(n-1\right)}}}$$

The admittance, exponent, and resistance of the constant phase elements are defined as Q, n, and R, respectively.

It can be observed the R_ct_ value increased with the addition of AlN from 3.6 k to 30 kΩ cm^2^; however, the double-layer capacitance dwindled from 2.4 to 0.6 μF as a result of the occurrence of the inactive AlN particles within the aluminum matrix that obstructed the ingress of the hydrated chloride species.

**Figure 15:**
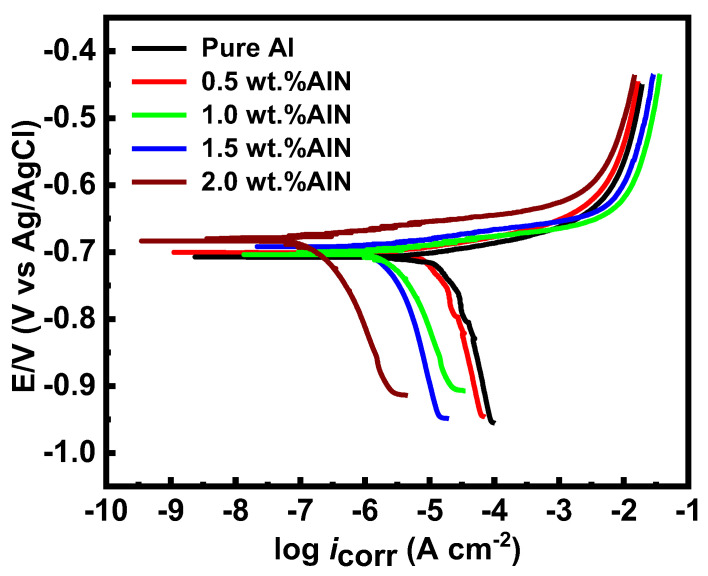
Potentiodynamic polarization curves of the Al-AlN composites using different volume percentages of AlN (0, 0.5, 1.0, 1.5, and 2.0) in 3.5% NaCl solution.

Figure 15 shows the potentiodynamic polarization curves of the aluminum matrix in 3.5 wt.% NaCl solution. The relevant electrochemical parameters are displayed in Table 3, such as the corrosion potential (E_corr_), anodic Tafel slopes (β_a_), cathodic Tafel slopes (β_c_), and corrosion current density (i_corr_).

The corrosion rate was calculated using the following formulation [48]:

(4) $$CR=\frac{i_{corr}.K.EW}{dA}$$

where CR is the corrosion rate, in mm y^−1^; i_corr_ is the corrosion current, in amps; K is the constant (3272); EW is the equivalent weight, in grams/equivalent; d is the density, in grams cm^−3^; and A is the exposed surface area of the Al-AlN composites, in cm^2^.

Aluminum degrades in the presence of the chloride species (Cl^−^ ions), shifting the potential in the –Ve direction according to the following reaction:

(5) $$Al={Al}_{3}+{3e}^{-}$$

The oxygen reduction that occurred on the Al composite matrix over the cathodic reaction is given by the following formulas:

(6) $$\frac{1}{2}O_{2}+H_{2}O+e^{-}=OH_{ads}+OH^{-}$$

(7) $$O_{2}+2H_{2}O+{4e}^{-}=4OH^{-}$$

According to the point defect model (PDM), when the flux of cation vacancies (j_ca_) > the rate of annihilation of cation vacancies (j_m_), the cation vacancies on the cation sublattice of the barrier layer $\left(V_{M}^{x^{\prime }}\right)$, will be concentrated at the metal/bilayer interface. Therefore, a blister will result from the residual barrier layer detaching from the base metal at a certain concentration of the cation vacancy. Thus, $V_{ö}$ will be constructed on the metal surface due to the barrier layer’s inability to grow. It is possible that the oxygen vacancies $V_{ö}$ generated at the bi/electrolyte interface will autocatalytically react with more Cl^−^ ions, thereby creating more oxygen and cation vacancies. At highly susceptible locations with the highest diffusivities for cation vacancies, the generated cation vacancies could transfer to the Al/bi-layer interface and slowly accumulate, leading to the breakdown of the protective layer. The AlN particles act as a physical barrier between the corrosive environment and the aluminum matrix, reducing the exposure of the underlying layer of aluminum to corrosive species by reducing the presence of vacancies at the metal/bi-layer interface. This can decrease the likelihood of corrosion initiation and slow down the corrosion rate and promote the formation of a more stable and protective oxide layer. It is noteworthy that the corrosion rate of Al significantly decreased from 0.16 to 0.005 mm y^−1^ with the inclusion of 2.0 vol.%AlN.

## 4. Conclusions

This study demonstrated the effectiveness of utilizing a microwave sintering furnace for heat-treating prepared Al-AlN composite samples using a powder metallurgy approach. The surface inspection proved the presence of the Al matrix and its AlN reinforcement with the presence of a few AlN agglomerations found at higher concentrations. The compositional analysis provided better insight into the distribution of the AlN nanoparticles within the Al matrix. The structural analysis confirmed the purity of the formed Al-AlN composite patterns, defining their crystallinity and intensity in relation to the incorporated amount of AlN reinforcement. The density of the fabricated composites was strongly influenced by the nanosized nature of the AlN particles and their added volumetric amount in the composites. The micro- and nanohardness values exhibited promising outcomes for the inclusion of AlN nanoparticles in an Al base metal, verifying the worthiness of the chosen AlN reinforcement material. The compressive strengths of the composites and Al billet were measured and found to gradually decrease because of the occurrence of AlN agglomerations. A shear mode fracture was observed extensively in the Al-AlN composites under applied compressive stress. The inclusion of AlN particles in the Al matrix considerably reduced the corrosion rate from 0.16 mm y^−1^, for the pure Al, to 0.005 mm y^−1^.

## Figures and Tables

**Table 1:** Content ratios of the Al-AlN composites.

Sample No.	Sample Name	Volume Fraction of AlN (vol.%)
1	A1	0
2	A2	0.5
3	A3	1
4	A4	1.5
5	A5	2

**Table 2:** The electrochemical parameters from the Nyquist plots for the Al-AlN composites using different weight percentages of AlN (0, 0.5, 1.0, 1.5, and 2 vol.%) in 3.5% NaCl solution.

Sample	R_ox,_ kΩ cm^2^	R_ct,_ kΩ cm^2^	C_dl1_ μF	C_dl2_ μF
Pure Al	0.645	3.6	2.2	2.4
0.5 vol.%AlN	0.712	5.1	1.8	1.6
1.0 vol.%AlN	1.5	15	1.4	1.3
1.5 vol.%AlN	3.4	19	1.1	0.8
2.0 vol.%AlN	4.3	30	0.9	0.6

**Table 3:** Electrochemical parameters of the aluminum matrix before and after inclusion of AlN.

Sample	βa (V·decade^−1^)	βc (V·decade^−1^)	i_corr_ (µ A cm^−2^)	Corrosion Rate (mm y^−1^)
Pure Al	0.0141	0.151	8	0.16
0.5 vol.%AlN	0.0137	0.087	4.1	0.082
1.0 vol.%AlN	0.0131	0.066	2.5	0.05
1.5 vol.%AlN	0.0121	0.027	1.2	0.02
2.0 vol.%AlN	0.0111	0.012	0.23	0.005

## Data Availability

The raw data supporting the conclusions of this article will be made available by the authors on request.
